# Requirements and Implementation Challenges of Automated Outbreak Detection Systems in the European Union: Mixed Methods Study

**DOI:** 10.2196/96576

**Published:** 2026-09-15

**Authors:** Paulina vom Felde genannt Imbusch, Ann Christin Vietor, Inessa Markus, Michaela Diercke, Alexander Ullrich

**Affiliations:** 1 Department of Infectious Diseases Epidemiology, Robert Koch Institute, Seestr. 10, Berlin, State of Berlin, 13353, Germany, 49 30187540

**Keywords:** public health surveillance, communicable diseases, disease outbreaks, algorithms, public health informatics

## Abstract

**Background:**

Automated outbreak detection can enhance infectious disease surveillance by enabling early identification of outbreaks and supporting timely public health measures. However, information on its current use by national public health institutes remains limited.

**Objective:**

This study aimed to provide an updated and extended overview of automated outbreak detection use in the European Union and the United Kingdom by (1) assessing current demand, (2) examining the availability of key prerequisites within existing surveillance systems, and (3) identifying challenges and requirements for implementation.

**Methods:**

A mixed methods approach was applied as part of the Joint Action UNITED4Surveillance. Data were collected between April 2023 and January 2024 through an online survey sent to 25 countries, an in-person workshop with experts from 16 countries, and follow-up meetings with participating national public health institutes. Additional information for selected countries was obtained from the literature. Data were analyzed descriptively.

**Results:**

Twenty-one countries completed the survey. In total, 33.3% (n=7) of the countries had established automated outbreak detection systems, 19% (n=4) were planning implementation, and 47.6% (n=10) participated in a pilot project. All surveyed countries reported acting on detected surveillance signals. Most had suitable surveillance infrastructure, including case-based data (n=20, 95.2%), daily reporting (n=21, 100%), and at least 3 years of historical data (n=21, 100%). Main barriers to implementation included limited funding (n=15, 71.4%), insufficient IT capacity, and data quality issues (n=9, 42.9%). Despite heterogeneity in methods and system design, outputs and user requirements were largely similar across countries, with needs for flexible outputs, stratification, and user-friendly interfaces.

**Conclusions:**

While the specific methods in existing automated outbreak detection systems differ, overall demands and outputs are similar, suggesting that a single tool could serve multiple countries. European Union–funded Joint Actions may help address some of these barriers by supporting collaborative tool development and knowledge exchange.

## Introduction

Timely detection of infectious disease outbreaks, enabling targeted investigations and control measures, is a core objective of public health surveillance [1,2]. Outbreak detection relies on identifying case numbers that exceed the expected threshold, typically recognized through accumulated case reports of notifiable diseases or outbreak notifications from reporting entities. With the growing availability of electronic case reports, automated outbreak detection has gained significant importance, offering a systematic approach to identify outbreaks [1,3,4].

Outbreak detection refers to the process of identifying unusual increases in case numbers by comparing the current epidemiological situation with a baseline, usually established from historical or geographical data. Statistical methods estimate the expected number of cases and compute a threshold. When observed values deviate significantly from these expectations, surpassing the threshold, signals are generated and often presented in tables, visualizations, or descriptive reports. These signals necessitate epidemiological investigations to determine whether they represent potential outbreaks or false positives [5].

Various methods for outbreak detection exist and have evolved over time [6-8]. Among the most widely used are the method by Farrington et al [9] and adaptations by Noufaily et al [10], which use generalized linear models to predict the expected number of cases accounting for seasonality, time trend, and overdispersion. Building on these methods, we can distinguish a hierarchy of components: statistical methods underlie tools that operationalize them, and some of these tools allow for the automated analysis of complete surveillance datasets. Here, automation means performing outbreak detection across relevant dimensions such as region, age, or subtype rather than sequentially inspecting individual strata and time series. Together with the necessary infrastructure, standard operating procedures, and trained personnel, these tools constitute comprehensive automated outbreak detection systems (AODSs).

Despite methodological advances, information on AODS implementation in the European Union (EU) is limited. The most recent overview from 2010 reported 6 institutes in 5 countries (Denmark, Germany, the Netherlands, Sweden, and the United Kingdom) using automated detection for routine surveillance. On the basis of the existing AODSs and practical experiences, recommendations were drawn, including the regular evaluation of AODSs and aiming for a user-friendly approach directed at epidemiologists [11,12]. Additionally, a 2017 workshop involving stakeholders from 17 institutions across the EU emphasized the exchange of data and experiences across countries, the need for visual outputs for users, and assessment and comparison of detection methods [13].

The EU4Health program is the EU’s funding instrument for strengthening public health systems and preparedness. Within this program, Joint Action UNITED4Surveillance (U4S) brings together national public health institutes (NPHIs) across Europe to improve integrated infectious disease surveillance, including strengthening capacities for outbreak detection and response. One aim of U4S was to identify the requirements of EU countries for effective outbreak detection [14]. Understanding these needs and current practices is a necessary first step before developing and implementing new AODSs. While the project also involved developing and piloting an open-source R Shiny app for automated outbreak detection by multiple NPHIs, this study provides an updated and extended overview of automated outbreak detection use in EU countries and the United Kingdom. The analysis encompassed (1) assessing current demand for automated outbreak detection, (2) examining the availability of necessary prerequisites of surveillance systems, and (3) identifying challenges and requirements for running AODSs.

## Methods

### Overview

A mixed methods approach was used, combining a survey, a workshop, and online meetings as part of the U4S project. The United Kingdom and France did not participate in the survey but were included as additional case studies because the 2010 overview and the workshop described in the Introduction section identified these countries as operating relevant AODSs [11,13]. In the 2010 overview, UK systems were reported separately for England, Wales, and Northern Ireland. Therefore, our analysis distinguishes these jurisdictions where relevant. Reporting of this work followed the SRQR (Standards for Reporting Qualitative Research) [15].

### Questionnaire Development

A questionnaire was drafted with the goal of identifying gaps and needs from the perspective of epidemiology and data science. Respondents were asked to select one national surveillance system either in current use or considered most suitable for automated outbreak detection. The questionnaire had two sections: (1) description of public health surveillance systems, including types, legal frameworks, disease groups, data sources, aggregation level, reporting frequency, indicators, geographical resolution, and historical data availability; and (2) automated outbreak detection capacities, covering resource availability; signal follow-up; and details of existing methods, resources, and outputs.

The draft was reviewed by 6 project partners and a contact point at the European Centre for Disease Prevention and Control and revised accordingly. The final version (Multimedia Appendix 1) contained 33 questions, of which 22 were mandatory, primarily single- or multiple-choice rankings and free-text fields.

### Survey Administration

The survey was created using the EUSurvey platform and distributed on April 17, 2023, by the U4S coordinator to designated contact points of the 25 countries participating in the project. These contact points were representatives of the participating NPHIs or partner institutions and served as the official coordinators for project activities within their respective countries. They were asked to coordinate questionnaire completion with relevant national experts, including epidemiologists and data scientists, where necessary, and submit one consolidated response describing the national surveillance system considered most suitable for automated outbreak detection.

### Workshop

An on-site workshop was held in May 2023 at the Robert Koch Institute in Berlin, Germany, as part of the U4S project. Participants were invited from institutions participating in the U4S project, with active partners involved in the outbreak detection work package encouraged to attend in person. To ensure balanced representation across participating institutions, attendance was limited to a maximum of 2 representatives from active outbreak detection partners and 1 representative from other beneficiary institutions. In total, 30 public health experts, epidemiologists, and data scientists from NPHIs representing 16 EU countries participated. The workshop aimed to discuss survey findings, identify relevant use cases, and plan the pilot phase. Participating countries presented existing AODS and surveillance system overviews. Participants were divided into 2 breakout groups. Data scientists worked on defining outbreak detection tools achievable within the following 12 months, including requirements, vision, data input and output, and handling (operation, users, interfaces, and reporting). Epidemiologists, as future tool users, worked on outlining the ideal tool, specifying intended functionality, data sources, pathogens, use cases, and operational aspects.

### One-on-One Meetings and Calls

In January 2024, structured one-on-one meetings were conducted with the 10 piloting countries participating in the tool implementation phase. Between April 2024 and November 2024, these countries piloted the outbreak detection tool by integrating their national surveillance data into the required input format, using the tool in routine surveillance, providing feedback, reporting bugs, suggesting additional requirements, and participating in the tool evaluation. Meetings included the national project representatives and technical experts involved in surveillance and data management. Discussions followed a predefined agenda covering country-specific surveillance structures, administrative divisions, data availability, and functional requirements for the outbreak detection tool. Additional informal online meetings conducted between 2023 and 2024 further supported the collection and clarification of tool requirements.

### Data Analysis

Survey responses were exported from EUSurvey and analyzed using R (version 4.4.0; R Foundation for Statistical Computing) [16]. As this study aimed to describe the characteristics of national surveillance systems and automated outbreak detection capacities across participating countries, analyses were descriptive. Categorical variables are presented as counts and percentages where appropriate, whereas free-text responses were reviewed and summarized thematically to capture common approaches and country-specific characteristics.

### Data Verification

Survey results were first presented during the workshop in May 2023, allowing participants to review and validate the data. During a project webinar in June 2023, the survey and workshop results were shared with the entire project consortium, giving all survey respondents the opportunity to verify their data.

### Ethical Considerations

No formal ethics committee approval was sought because this study did not involve patients, clinical interventions, or the collection of sensitive personal data, and findings were analyzed and reported only in aggregated country-level form. The processing of participants’ contact information was reviewed in consultation with the Robert Koch Institute’s Data Protection Office and conducted in accordance with the General Data Protection Regulation (Regulation [EU] 2016/679) and the German Federal Data Protection Act (*Bundesdatenschutzgesetz*).

Participants provided electronic informed consent before completing the survey. Names and email addresses were used solely for study administration and were not included in the analysis. All results are presented in aggregated form, ensuring that individual participants cannot be identified.

## Results

### Overview

**Figure 1.**
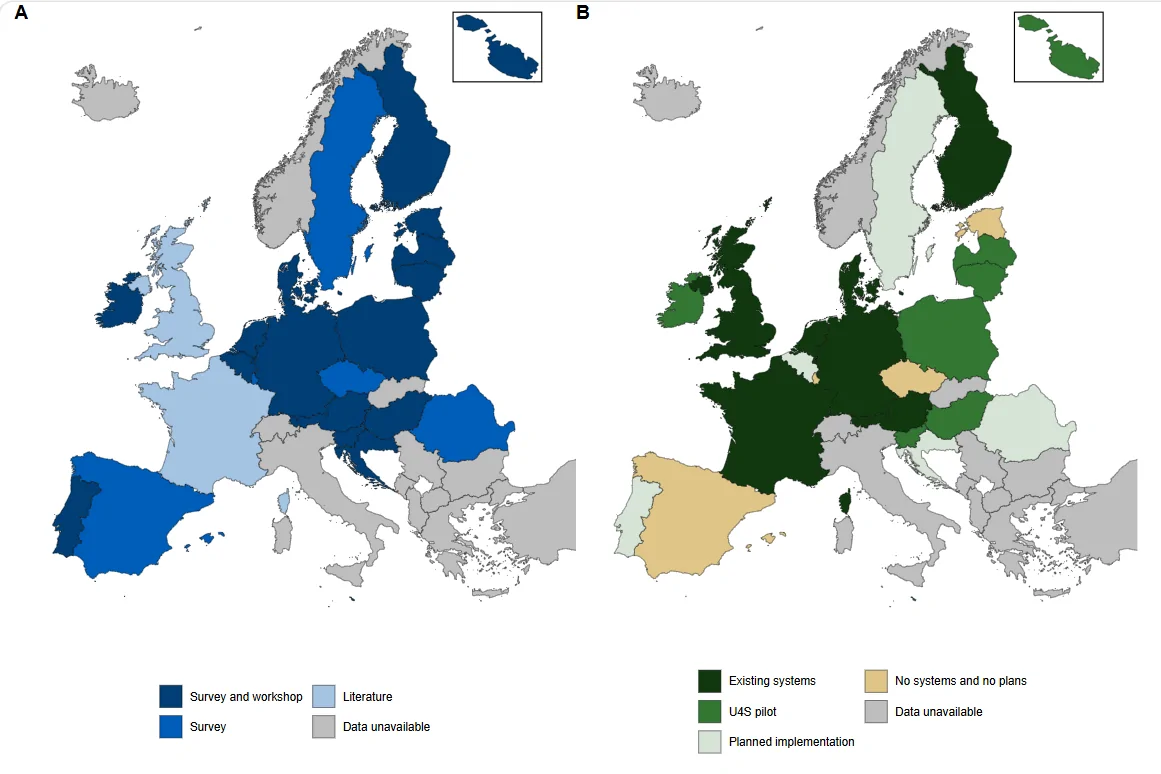
Map showing the current status of automated outbreak detection systems (AODSs) in the European Union (EU) and the United Kingdom and the corresponding source of information (2024). (A) Source of information used to assess the status of AODSs across the EU and the United Kingdom. All countries that participated in the workshop also completed the survey. The upper right inset shows Malta. (B) Countries are categorized by their current level of implementation of AODSs. In cases in which countries meet the criteria for multiple categories (eg, Finland, Denmark, and the Netherlands, which have existing AODSs and are also UNITED4Surveillance [U4S] pilot partners), the higher level of implementation is displayed. “U4S pilot” refers to those countries that used the automated outbreak detection tool developed within U4S in a pilot phase from April 2024 to December 2024. The upper right inset shows Malta.

The analysis included 26 countries, comprising 25 (96.2%) EU member states and the United Kingdom. Of the 25 U4S consortium members invited to participate in the survey, 21 (84%) completed it. France did not respond. The United Kingdom was not invited because it was not part of the U4S consortium. The largest group of countries (16/26, 61.5%) contributed data through the survey and participation in the workshop. A total of 19.2% (5/26) of the countries participated in the survey only; data for 7.7% (2/26) of the countries were obtained solely from the literature; and for 11.5% (3/26) of the countries, no data were available (Figure 1). Most survey respondents (19/21, 90.5%) were associated with NPHIs, and the remainder were associated with ministries of health (2/21, 9.5%).

### Current Use of AODSs in EU Countries

On the basis of the data collected from EU countries and the United Kingdom, 26.9% (7/26) of the countries had existing AODSs in place. A total of 38.5% (10/26) of the countries participated in the U4S pilot project, whereas 15.4% (4/26) were planning to implement such AODSs. Another 15.4% (4/26) had neither AODSs nor plans for implementation, and for 11.5% (3/26) of the countries, no data were available (Figure 1). Country-specific information on data sources and implementation status for each of the 26 countries is also provided in Multimedia Appendix 2.

### Identified Need for Outbreak Detection

Beyond the current level of implementation, our survey examined how countries responded to signals generated from surveillance data, whether detected manually or automatically. While a diverse level of implementation across the EU was observed, all countries that completed the survey (21/25, 84%) stated that they would act on a signal identified through their surveillance data, whether from manual inspection or automated analysis. Typically, this response involved verification of the signal, followed by the initialization of an investigation, a risk assessment, further communication, and introduction of measures if needed. In most cases (14/21, 66.7%), the corresponding NPHI received feedback on actions triggered by a signal via outbreak reports (6/14, 42.9%), via direct communication with the investigation authorities (3/14, 21.4%), or by observing the performed public health action (2/14, 14.3%). In one case, investigations were conducted directly by the NPHI. In many countries (11/21, 52.4%), feedback on actions was collected systematically and often also used for evaluation (8/11, 72.7%). In addition, piloting countries grew from initially 7 to 10, reflecting the high interest in the tool.

### Identified Challenges to Implementing AODSs

When asked about main challenges in implementing AODSs, lack of sustainable funding and resources was indicated as the primary obstacle (15/21, 71.4%). This was followed by data quality (9/21, 42.9%) and legal mandate issues (5/21, 23.8%). In one country, data quality specifically referred to limited data availability and reporting delays. A total of 9.5% (2/21) of the countries reported no barriers, whereas another 9.5% (2/21) reported “other” issues. Of these countries, one respondent (Sweden) noted that an automated detection system had previously been implemented in the surveillance system but had not yet been reimplemented following a system upgrade in 2022. The other country (Lithuania) cited concerns about potential functional incompatibilities between systems, referring to technical issues that can cause delays or inconsistencies when data are synchronized across platforms before integration into the national eHealth system.

**Figure 2.**
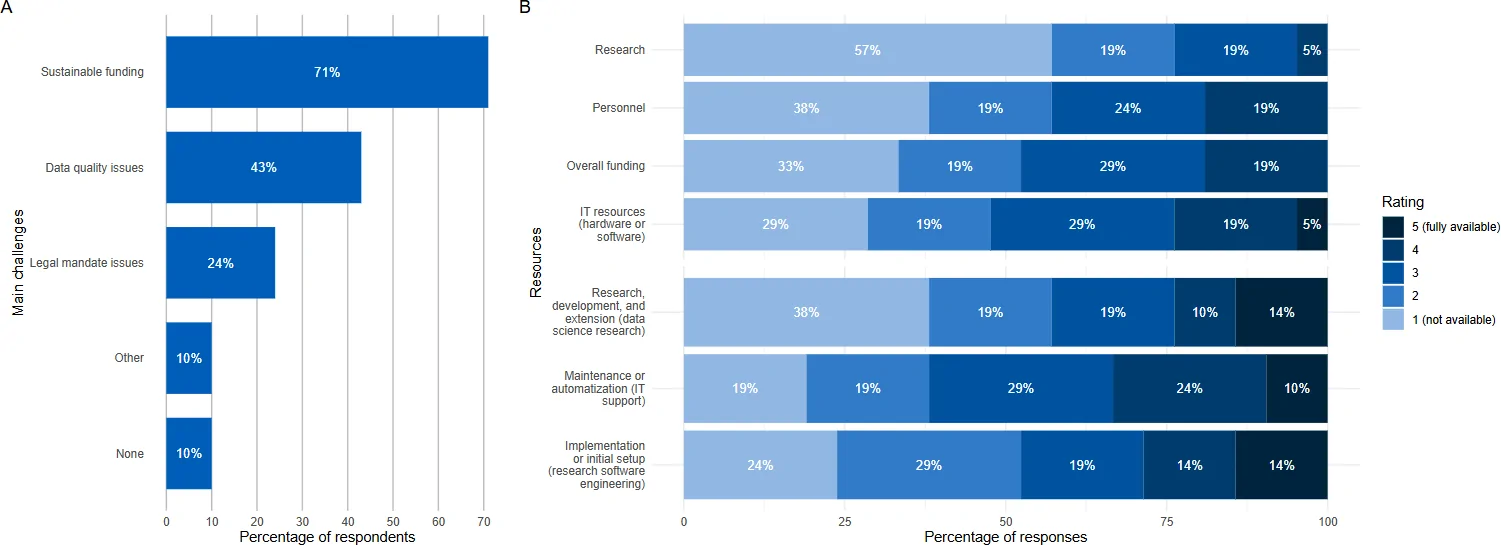
Reported challenges and resources for implementing automated outbreak detection systems (AODSs) in the European Union based on a survey of national public health institutes in 2023. (A) Main challenges reported by countries in implementing AODSs. Multiple answers per respondent were possible. (B) Aggregated ratings (1-5) of funding availability and IT competences to run and support implementation. Higher scores indicate greater perceived availability.

Regarding funding, respondents reported particularly limited funding for research capacity, specifically staff knowledge and time for research activities (12/21, 57.1%), personnel (8/21, 38.1%), overall funding (7/21, 33.3%), and IT resources such as hardware and software (6/21, 28.6%). Similarly, no funding was available for IT competences, including research software engineering for implementation (6/21, 28.6%), IT support for maintenance and automatization (6/21, 28.6%), and data science expertise for development and extension (8/21, 38.1%; Figure 2).

### Surveillance System Prerequisites

Despite these implementation barriers, many countries already possessed surveillance infrastructures that provided favorable conditions for AODS implementation. The performance of AODSs primarily relies on the information available in the surveillance system, including historical data used to establish baselines, detect trends, and adjust for seasonality [5]. Almost all respondents stated that data were reported in a case-based format (20/21, 95.2%) and on a daily basis (19/21, 90.5%) at the national level. A total of 57.1% (12/21) stated that additional personally identifiable data (eg, addresses, date of birth, and personal identity number) were available. Furthermore, all countries (21/21, 100%) had at least 3 years of historic data available, and 38.1% (8/21) had historic data for 20 years or more.

All countries (21/21, 100%) received information on age, region, pathogen, and sex of infectious disease cases through their surveillance systems at the national level. These are the most common stratification dimensions in AODSs and allow for extensive investigation. Most countries also had additional information on outcomes such as death (19/21, 90.5%), hospitalization (17/21, 81%), and date of symptom onset (17/21, 81%) or specific symptoms (14/21, 66.7%), which would allow for further assessment of signals regarding the severity or even outbreak detection on different indicators (eg, number of hospitalizations). In many countries, information on vaccination status (15/21, 71.4%), risk factors (11/21, 52.4%), or exposures (15/21, 71.4%) was available, which would enable further disease-specific analyses.

### Identified Use Cases for AODSs

Given these surveillance capacities, we next explored the disease areas and scenarios in which countries saw the greatest potential for AODSs. Identifying potential use cases for automated outbreak detection is essential for designing an effective and widely used AODS. This includes specifying data input formats, defining user-configurable input options such as filters and parameters, and ensuring that analysis and outputs are actionable and relevant for various disease surveillance systems [17].

The survey collected information on disease groups targeted by the surveillance systems, revealing potential use cases for AODSs and groups that could benefit the most from these systems. Food- and waterborne diseases (19/21, 90.5%), airborne diseases (18/21, 85.7%), and vector-borne and zoonotic diseases (17/21, 81%) were the most common disease groups. At the workshop, gastroenteritis and respiratory diseases emerged as the main use cases, with stronger interest in gastroenteritis. Accordingly, pilot use cases focused mainly on gastrointestinal pathogens, with one other country selecting group A streptococci.

### Identified Requirements for AODSs

The identified requirements for AODSs drawn from one-on-one meetings and calls with the piloting countries showed significant overlap despite different organizational and geographical structures. Key needs included flexible output formats, adaptability for pandemic periods, and various filtering and stratification options. The systems should also provide information on cases related to already known outbreaks; enable extraction of detailed case line lists; support different temporal aggregations, such as daily, weekly, and monthly; and offer multiple language options. Several countries also highlighted the need to accommodate whole-genome sequencing data within future outbreak detection tools. The workshop discussion highlighted that signals were managed by different experts across countries with varying levels of IT expertise, underscoring the need for user-friendly, accessible, and adaptable tools. These requirements highlight the need for a flexible system that can accommodate diverse user needs effectively.

### Current AODSs Implemented by Countries

Table 1 presents an overview of current AODSs implemented in 26.9% (7/26) of the countries. The results are presented in 2 sections highlighting the differences in type and depth of information between countries that participated in the U4S project and France and the United Kingdom, for which only literature-based data were available.

**Table 1.** Characteristics of automated outbreak detection systems for infectious disease surveillance in 5 countries from the UNITED4Surveillance project (survey data) and France and the United Kingdom (literature; 2024).

Country	Pathogens and surveillance data	Methods	Implementation and automation	Reporting and frequency	Remarks
Austria	*Campylobacter *and *Salmonella*; case data from the national reference laboratory	Farrington Flexible algorithm and CUSUM^a^	R, *surveillance* package; fully automated	Weekly report	COVID-19–era hiring added data scientist capacity; plan to use data from the national electronic reporting system and extend to all notifiable diseases
Denmark	Approximately 20 assorted notifiable diseases, *Campylobacter*, and COVID-19; laboratory data from the national microbiology database	Threshold rules for approximately 20 diseases; Farrington Flexible algorithm; in-house method for COVID-19 prevalence levels	SQL+R+SAS; *surveillance* package; automation varies	Daily automatic email alerts (approximately 20 diseases); weekly ad hoc R reports or web figure	No single framework; evaluation pending; initiative to unify
Finland	Influenza; cases from Register of Primary Health Care Visits	MEM^b^	R, *mem* package; fully automated	Daily website updates on case numbers, thresholds, and epidemic levels	MEM since 2015; focused on influenza; plan to monitor other infectious diseases with data from the National Infectious Diseases Register
Germany	Approximately 30 diseases; national case reports from German surveillance database	Farrington Flexible algorithm	R pipeline, *surveillance* package; fully automated	Comprehensive weekly report (maps, charts, and tables)	Extensive coverage of notifiable pathogens
The Netherlands	Approximately 40 diseases; case-based data in the RIVM^c^ database	HMM^d^+spline seasonality [18]	R Shiny dashboard (“survboard”); ad hoc computation during use of dashboard	Interactive dashboard; frequency dependent on use	Real-time geographic, temporal, and demographic views; plan to use WGS^e^ data for outbreak detection with the new Shiny app
France (SurSaUD)—syndromic	Influenza+11 other syndromes; ED^f^ and SOS Médecins visits+mortality (approximately 700 EDs and 60 GP^g^ units); >90% ED coverage	Ensemble of GLMs^h^, robust GLMs (weighted), and HMM [19]	Daily R scripts at Santé publique France; alarms fully automated; human validation of alert levels	Daily national dashboard and weekly reports	Performance benchmarked comparing ensemble vs individual methods and application to different data sources
United Kingdom—England (syndromic)	Comprehensive coverage of syndromes; NHS^i^ 111 calls, GP consultations, and ED attendances	RAMMIE^j^ [20]	Automated Stata script; daily	Daily internal alerts and public weekly bulletins	RAMMIE demonstrated high sensitivity (approximately 92%) and specificity (approximately 99%); adjusts for weekday and holiday effects
United Kingdom—England and Wales (laboratory isolates)	>3300 distinct organisms; HPA^k^ surveillance data	Farrington Flexible algorithm (quasi-Poisson GLM)	R scripts (*surveillance* package); fully automated	Weekly reports emailed to national and regional epidemiologists	One of Europe’s oldest automated systems; continuously refined

a CUSUM: cumulative sum control chart.

b MEM: moving epidemic method.

c RIVM: National Institute for Public Health and the Environment.

d HMM: hidden Markov model.

e WGS: whole-genome sequencing.

f ED: emergency department.

g GP: general practitioner.

h GLM: generalized linear model.

i NHS: National Health Service.

j RAMMIE: rising-activity multilevel mixed-effects indicator emphasis.

k HPA: Health Protection Agency.

The 19.2% (5/26) of countries participating in the U4S project (Austria, Denmark, Finland, Germany, and the Netherlands) all used routinely collected notifiable infectious disease surveillance data and made use of R-based workflows, including the *surveillance* R package [21]. They differed, however, in the number and type of pathogens monitored, the specific outbreak detection method applied, the degree of automation, and the type of reporting. While some countries used their AODSs for individual pathogens, others covered a broad range of notifiable diseases. Despite this heterogeneity, all systems generated near–real-time outputs. Several countries were currently working to expand pathogen coverage, integrate national surveillance data, or harmonize their workflows.

France and the United Kingdom operated long-established R-based AODSs across diverse national data streams and used established R-based analytic frameworks. Both countries had comprehensive coverage of syndromes (France and the United Kingdom) or pathogens (United Kingdom) and automated R scripts run daily or weekly, producing internal alerts, dashboards, and bulletins. The systems in France and the United Kingdom have been established for many years; reflect a high level of methodological maturity, including systematic evaluations; and offer guidance for further development and application of AODSs in other countries.

## Discussion

### Principal Findings

This study provides an updated overview of the current landscape of AODSs in the EU and the United Kingdom by assessing current demand for automated outbreak detection, evaluating the availability of surveillance system prerequisites, and identifying barriers and requirements for implementation. Our findings reveal a heterogeneous state of implementation, with only 26.9% (7/26) of the countries included in the analysis operating AODSs. However, our results also highlight important opportunities for broader adoption, as surveillance systems in many countries already meet key data-related prerequisites for automated outbreak detection, including the availability of case-based, frequently reported, and sufficiently extensive historical surveillance data.

Compared to the 2010 overview, which outlined AODSs in Denmark, England, Germany, the Netherlands, and Sweden, our findings show that the overall landscape has changed little [11]. Most of these countries continued to operate AODSs, except Sweden, which discontinued its system following the implementation of a new surveillance system. Austria and Finland were the only countries that had introduced new systems: Austria’s enhanced capacities during the COVID-19 pandemic may reflect increased prioritization of AODSs enabled by the additional resources made available during the pandemic. In contrast, Finland’s adoption using the moving epidemic method focused more on retrospective trend analysis than on real-time signal detection. The findings also suggest that high-resource countries are more likely to establish outbreak detection systems but maintaining them can become challenging when substantial changes in surveillance systems occur. This limited progress appears to reflect persistent structural barriers rather than a lack of demand as respondents consistently identified sustainable funding, IT capacity, data quality, and legal mandates as the main obstacles to implementation.

Deficits in software engineering, IT support, and data science restrict the ability of NPHIs to develop and sustain systems. These constraints help explain why, despite clear interest, only a minority of countries currently operated AODSs. Similarly, the 15.4% (4/26) with neither an operational AODS nor plans for implementation reported barriers including limited funding, data quality, legal mandates, and IT capacity, with 2 also citing low political priority or a limited need due to a small number of notifications. At the same time, surveillance environments are evolving. An increasing number of countries are routinely generating whole-genome sequencing data as part of surveillance activities, which introduces new requirements for outbreak detection tools, particularly with regard to data input formats, interoperability, and the ability to integrate sequencing information alongside epidemiological data.

Our survey and pilot project further indicate growing momentum toward automation. In total, 38.5% (10/26) of the countries actively piloted the developed tool, demonstrating both the technical feasibility of implementation and the willingness of NPHIs to integrate methods into routine practices. In parallel, all survey respondents (21/21, 100%) confirmed that they would act on signals detected in their surveillance data. This finding indicates that the principle of responding to signals is firmly embedded in national public health practice. Structured procedures for response were in place in most countries (18/21, 85.7%), yet the way feedback was collected and integrated varied considerably. Reliance on manual verification may cause delays and inconsistencies, whereas greater automation could enable timelier signal identification, more consistent risk assessment, and systematic feedback.

Moreover, the results show significant potential for implementing new outbreak detection systems as many countries reported having extensive historical data, often exceeding 8 years, which is crucial for establishing baselines and trend analysis. Identifying specific use cases such as gastrointestinal pathogens highlighted the practical value of AODSs in routine surveillance tasks. Another potential application of new methods such as spatial clustering at the address level arises from the availability of certain personally identifiable data. The ability to incorporate such data allows for more precise mapping and detection of patterns.

Experience from pilot and established systems showed substantial alignment in outbreak detection needs across the EU. Despite differences in epidemiological contexts and organizational structures, system requirements showed considerable overlap. Pilot countries highlighted needs such as flexible output formats, temporal aggregation, filtering, and multilingual support, whereas established systems used different statistical methods and infrastructures yet generated largely comparable outputs. The convergence of outputs despite methodological differences suggests that key outputs, including signals, visualizations, maps, and reports, could be standardized. These shared requirements indicate that a single flexible tool could accommodate diverse national contexts, meeting practical needs without requiring separate systems for each country. The widespread use of the *surveillance* package is a good example of the utility of open-source tools developed by the public health community.

### Limitations

Our study has some limitations. First, the survey focused on one surveillance system per country, which might not fully capture the use of AODSs across all national surveillance systems. Second, varying interpretations of automated outbreak detection could have influenced reported use, including differences between signal detection and data visualization. Third, reliance on self-reported data may have introduced bias or overestimation, with response depth varying by how extensively experts were consulted. Fourth, workshop participation was limited to 30 experts from 16 countries, not representing all EU contexts. Fifth, while the systems in the United Kingdom and France were still operational, the data may not reflect all subsequent updates. Sixth, saturation was not formally assessed or used as a criterion for determining the extent of data collection. Countries were predefined by their involvement in their respective U4S project activities. The qualitative components were used to complement and contextualize the country-level survey findings and further explore requirements for AODSs. Despite these limitations, this study provides valuable insights into existing practices and priority areas for improvement.

### Future Perspectives and Conclusions

AODSs offer the potential to enhance routine surveillance of infectious diseases across EU countries. However, the results of this study indicate that resource limitations and technical barriers continue to hinder their wider implementation. Broader adoption of flexible, open-source solutions could accelerate cross-country collaboration and promote more harmonized outbreak detection in the EU. The experience across countries with different surveillance capacities further suggests that AODSs can be adapted to diverse settings, although their implementation in more resource-limited contexts will depend on the availability of reliable surveillance data, digital infrastructure, and sustained technical support.

These findings have broader implications for infectious disease surveillance within the EU. Increasing harmonization of outbreak detection approaches could facilitate knowledge exchange between countries, support more comparable surveillance practices, and reduce duplication of effort through the shared development and maintenance of open-source tools. Collaborative initiatives such as U4S may help bridge existing implementation gaps by bringing together expertise, building technical capacity, and fostering collaboration across countries.

Future work should focus on integrating the R Shiny app within the U4S project into local systems; expanding its use beyond the project phase; and refining methods, interfaces, and data integration. Broader adoption of such flexible, open-source solutions could accelerate cross-country collaboration and promote more harmonized outbreak detection in the EU. As automated systems can generate large numbers of signals with varying specificity, systematic evaluation in routine public health practice will be essential to assess their accuracy, usability, workload implications, and overall added value for infectious disease surveillance.

## Supplementary material

10.2196/96576 Multimedia Appendix 1 Survey on needs and gaps analysis of automated outbreak detection tools for routine surveillance data.

10.2196/96576 Multimedia Appendix 2 Overview of country-specific source of information and automated outbreak detection system implementation status across 26 countries, 2024.

10.2196/96576 Checklist 1 SRQR checklist.

## Abbreviations

AODS: automated outbreak detection system
EU: European Union
NPHI: national public health institute
SRQR: Standards for Reporting Qualitative Research
U4S: UNITED4Surveillance

## References

[1] Yuan M, Boston-Fisher N, Luo Y, Verma A, Buckeridge DL (2019). A systematic review of aberration detection algorithms used in public health surveillance. J Biomed Inform.

[2] Steele L, Orefuwa E, Dickmann P (2016). Drivers of earlier infectious disease outbreak detection: a systematic literature review. Int J Infect Dis.

[3] Buehler JW, Hopkins RS, Overhage JM, Sosin DM, Tong V, CDC Working Group (2004). Framework for evaluating public health surveillance systems for early detection of outbreaks: recommendations from the CDC Working Group. MMWR Recomm Rep.

[4] Branch-Elliman W, Sundermann AJ, Wiens J, Shenoy ES (2023). The future of automated infection detection: innovation to transform practice (part III/III). Antimicrob Steward Healthc Epidemiol.

[5] Zareie B, Poorolajal J, Roshani A, Karami M (2023). Outbreak detection algorithms based on generalized linear model: a review with new practical examples. BMC Med Res Methodol.

[6] Page ES (1954). Continuous inspection schemes. Biometrika.

[7] Hutwagner L, Thompson W, Seeman GM, Treadwell T (2003). The bioterrorism preparedness and response Early Aberration Reporting System (EARS). J Urban Health.

[8] Unkel S, Farrington CP, Garthwaite PH, Robertson C, Andrews N (2012). Statistical methods for the prospective detection of infectious disease outbreaks: a review. J R Stat Soc Ser A Stat Soc.

[9] Farrington CP, Andrews NJ, Beale AD, Catchpole MA (1996). A statistical algorithm for the early detection of outbreaks of infectious disease. J R Stat Soc Ser A Stat Soc.

[10] Noufaily A, Enki DG, Farrington P, Garthwaite P, Andrews N, Charlett A (2013). An improved algorithm for outbreak detection in multiple surveillance systems. Stat Med.

[11] Hulth A, Andrews N, Ethelberg S (2010). Practical usage of computer-supported outbreak detection in five European countries. Euro Surveill.

[12] Salmon M, Schumacher D, Burmann H, Frank C, Claus H, Höhle M (2016). A system for automated outbreak detection of communicable diseases in Germany. Euro Surveill.

[13] Ghozzi S, Ullrich A (2017). Bericht zum workshop “automatic detection of infectious disease outbreaks” [Article in German]. Robert Koch Institute.

[14] UNITED4Surveillance.

[15] O’Brien BC, Harris IB, Beckman TJ, Reed DA, Cook DA (2014). Standards for reporting qualitative research: a synthesis of recommendations. Acad Med.

[16] R Core Team (2024). R: a language and environment for statistical computing. R Foundation for Statistical Computing.

[17] Buckeridge DL (2007). Outbreak detection through automated surveillance: a review of the determinants of detection. J Biomed Inform.

[18] Vega T, Lozano JE, Meerhoff T (2013). Influenza surveillance in Europe: establishing epidemic thresholds by the moving epidemic method. Influenza Other Respir Viruses.

[19] Pelat C, Bonmarin I, Ruello M (2017). Improving regional influenza surveillance through a combination of automated outbreak detection methods: the 2015/16 season in France. Euro Surveill.

[20] Morbey RA, Elliot AJ, Charlett A, Verlander NQ, Andrews N, Smith GE (2015). The application of a novel “rising activity, multi-level mixed effects, indicator emphasis” (RAMMIE) method for syndromic surveillance in England. Bioinformatics.

[21] Salmon M, Schumacher D, Höhle M (2016). Monitoring count time series in R: aberration detection in public health surveillance. J Stat Softw.

