# Supplementary material for: Requirements and Implementation Challenges of Automated Outbreak Detection Systems in the European Union: Mixed Methods Study
Source: JMIR Public Health Surveill. 2026 Sep 15;12:e96576. doi: 10.2196/96576 (PMC13577613; doi:10.2196/96576)
Supplement: Supplementary file 1 [file publichealth-v12-e96576-s001.pdf]

UNITED4Surveillance

Needs and gaps analysis on automated outbreak detection tools for routine surveillance data

Fields marked with * are mandatory.

Introduction

UNITED4Surveillance

This survey is conducted within the Joint Action “United4Surveillance” as part of work package 2, task 2 on “Outbreak detection”.

Overall, this task focuses on improving algorithms for automated outbreak detection using routine surveillance data of infectious diseases. Benefits of introducing early outbreak detection tools include earlier detection of outbreaks, the detection of outbreaks that would not have been detected otherwise and thus reducing the further spread and harmful consequences. Yet, automated outbreak detection is only applied for a few of the available surveillance data streams, pathogens or pathogen groups.

This survey **aims** to get an overview of types of surveillance systems and automated outbreak detection methods currently used by EU/EEA member states and to identify gaps and needs for automated tools detecting outbreaks from routine surveillance data. The survey consists of two parts:

1. Public health surveillance system
2. Automated outbreak detection tools

Results will be used to identify common use cases for automated outbreak detection and guide the further development and deployment of tools in the European context.

Instructions

This survey is sent to selected focal points of countries that are part of the consortium of the Joint Action “United4Surveillance”.

- **Focal points are responsible for coordinating** the responses for their country and filling in the survey by **05.05.2023.**
- We encourage to fill out only one survey for each country (EU/EEA member states). If this is not feasible in your context please contact us.
- In case the survey needs to be completed by more than one expert (e.g. epidemiologist and data scientist), we strongly recommend that the focal point fills in the survey with the support of relevant national experts online.
- If you want to save your answers and return to them at a later time, please click on the right top button ‘save as draft’. **Please make sure to save the shown link.**
- Please use Internet Explorer, Mozilla Firefox or Google Chrome to fill out this survey. Using other browsers might cause compatibility problems.
- Once you have finished the survey, please click on ‘Submit’. You can save or print your contribution.
- The survey is also sent to the ECDC NFP Surveillance for information, awareness and facilitation if necessary.
- FAQ for participants on the use of the EU Survey Platform: https://ec.europa.eu/eusurvey/home/helpparticipants
- For questions about the survey itself and/or content of the survey, please contact vom-FeldeP@rki.de

Data privacy

This survey collects information from focal points of the Joint Action “United4Surveillance”. The name and e-mail address of the participants were provided by the contacted individuals themselves and therefore are already known within the consortium of the Joint Action “United4Surveillance”. Survey results will be evaluated on a country level and no individual personal information will be used.

☐ I have read and understand the above data privacy statement and I consent to participate in this survey.

Terminology

For a common understanding, the following terms used in the survey are explained below.

Automated outbreak detection is the process of comparing the current epidemiological situation to a baseline (usually based on historic and/or geographic data) using some form of outlier detection. It processes surveillance data in a way that demographic and geographic characteristics are considered. The detected outliers or signals are usually summarised in a table or descriptive report.

Case-based vs. aggregated data: Case-based data is surveillance data that is collected and processed in a way that each entry represents one case or report of an infection or disease event, whereas aggregated data summarizes the number of cases or reports in groups, e.g. by regions, age groups or other demographic information.

Historic data are surveillance data that were collected by the same surveillance system which will be used for the automated outbreak detection and that can be used to estimate a baseline against which the current surveillance data can be compared. These data may have to be adjusted for trends or shifts.

Library: A library for automated outbreak detection is a repository of programming code that provides functions for detecting signals (outbreak warnings) in surveillance data and possibly further functionality for reporting these results.

Tool/software: A software or tool for automated outbreak detection that takes surveillance data as input and provides the resulting outbreak warnings in the form of an automatically generated report or summary table. We will use the term “tool” for this survey.

Warning/Signal: A warning or signal is an outlier in a subset (e.g., a region or age group) of the surveillance data. An outlier is an anomalous number of cases or reports compared to a historic or geographic baseline. We will use the term “signal” for this survey.

General aspects

* Which country are you reporting for?

- AT - Austria
- BE - Belgium
- CY - Cyprus
- CZ - Czech Republic
- DE - Germany
- DK - Denmark
- EE - Estonia
- ES - Spain
- FI - Finland
- FR - France
- HR - Croatia
- HU - Hungary
- IE - Ireland
- IT - Italy
- LT - Lithuania
- LU - Luxembourg
- LV - Latvia
- MT - Malta
- NL - Netherlands
- NO - Norway
- PL - Poland
- PT - Portugal

- RO - Romania
- SE - Sweden
- SI - Slovenia

* Which organization do your work for?

- National Public Health Institute /National Public Health Authority
- Ministry of Health
- Other

If other, please specify.

Part 1: Public health surveillance system

We assume that in many EU/EEA member states several public health surveillance systems (e.g. mortality, pathogens, communicable diseases) exist. As it would be too extensive to assess every single surveillance system, we ask you to **choose one of your national public health surveillance systems**, for which an automated outbreak detection tool is already in place or which would be most suitable for automated outbreak detection tools in order to complete this survey.
Please complete the questions **only for the chosen surveillance system.**

* Thereby, the surveillance system can be one of three options. Please indicate which option applies to your chosen surveillance system.

- Surveillance system with existing automated outbreak detection tool/s
- Surveillance system with planned implementation of automated outbreak detection tool/s
- Surveillance system with no automated outbreak detection tool planned or in place, but where such tool/s would provide potential benefit

* What is the official name (full name and acronym) of the surveillance system?

* Approximately in what year was the system established?

The following questions only refer to the chosen national surveillance system you indicated above.

* Which type of surveillance system is it? Please choose multiple options if they are included in the surveillance system.

- Syndromic surveillance system
- Laboratory-based surveillance system
- Hospital-based surveillance system
- GP-based/ Primary care-based surveillance system

- [ ] Sentinel surveillance system
- [ ] Primary care-based surveillance
- [ ] Molecular-based surveillance system
- [ ] Event-based surveillance system

* Are the types of surveillance systems mandatory by law or voluntarily?

- ( ) Mandatory by law
- ( ) Voluntarily
- ( ) Not applicable
- ( ) Other

If other, please specify.

For which disease group is the surveillance system designed?

	Yes	No	Unknown
* Air-borne diseases (i.e. COVID-19)	( )	( )	( )
* Antimicrobial resistance (AMR)	( )	( )	( )
* Blood-borne diseases/ STI's (i.e. HIV)	( )	( )	( )
* Food- and water-borne diseases (i.e. Norovirus)	( )	( )	( )
* Healthcare-associated infections (i.e. Ventilator-associated Pneumonia)	( )	( )	( )
* Vector-borne diseases (i.e. Dengue fever)	( )	( )	( )
* Zoonotic diseases (i.e. Brucellosis)	( )	( )	( )

If other, please specify.

* Please indicate the source of the data, meaning the institution or entity generating the data which are reported to the (local) public health authorities (i.e. nuclei acid detection (i.e. PCR) of brucellosis in the laboratory and subsequent notification to the local health authority).

- [ ] Laboratories
- [ ] Hospital/medical personnel
- [ ] General Practitioner/primary care
- [ ] Blood bank
- [ ] Kindergarten/schools
- [ ] Care facilities/nursing homes
- [ ] Community
- [ ] Unknown
- [ ] Other

If other, please specify.

* What is the frequency of data reported to the national level?

- [ ] Daily
- [ ] Weekly
- [ ] Monthly
- [ ] Unknown
- [ ] Other

If other, please specify.

* How is data reported to your national level?

- [ ] Case-based
- [ ] Aggregated
- [ ] Unknown
- [ ] Other

If other, please specify.

If case-based, what information is available at national level?

- [ ] Anonymous information
- [ ] Pseudonymized information
- [ ] Personal identifiable data available
- [ ] Unknown
- [ ] Other

If case-based, please specify.

* Which information is reported routinely to the national level?

- [ ] Pathogen
- [ ] Disease
- [ ] Symptoms
- [ ] Date of symptoms onset
- [ ] Hospitalisation
- [ ] Possible exposure (e.g. epi-link)
- [ ] Risk factors
- [ ] Age
- [ ] Sex

- [ ] Geographical information (address/region)
- [ ] Laboratory method
- [ ] Date of laboratory confirmation
- [ ] Molecular and genomic typing
- [ ] Date of clinical diagnosis
- [ ] Vaccination status
- [ ] Death
- [ ] Unknown
- [ ] Other

If other, please specify.

* What is the geographical resolution of your data?

- [ ] Address
- [ ] District/neighbourhood
- [ ] Village/city
- [ ] County/province
- [ ] Region/state
- [ ] National/country
- [ ] Unknown
- [ ] Other

If other, please specify.

* How many years of historic data do you have electronically stored up to this point?

* Please briefly describe in a few sentences the most relevant features of the chosen surveillance system.

Part 2: Automated outbreak detection tools

The section "Resources for automated outbreak detection tool" should also be answered if your above described surveillance system does not have automated outbreak detection tools yet or is planning to implement them.

Resources for automated outbreak detection tools

Please answer the following three questions based on the resources that are currently available or can be made available for a foreseen future to implement automated outbreak detection tools.

How would you rate the availability for each of the following resources for running an automated outbreak detection tool for your surveillance system at this moment?
Please indicate from not available (1 star) to fully available (5 stars).

Overall funding	☆☆☆☆☆
Funding for personnel	☆☆☆☆☆
Funding for IT resources (hardware/ software)	☆☆☆☆☆
Funding fo research	☆☆☆☆☆

How would you rate the available IT competences to implement the automated outbreak detection tool at this moment?
Please indicate from not available (1 star) to fully available (5 stars).

Implementation/ Initial setup (Research Software Engineering)	☆☆☆☆☆
Maintenance/ Automatization (IT Support)	☆☆☆☆☆
Research/Development/Extension (Data Science Research)	☆☆☆☆☆

* Which main challenges do you currently have or anticipate to have when developing and implementing an automated outbreak detection tool?

- ☐ Data quality issues
- ☐ Legal mandate issues
- ☐ Sustainable funding/resources
- ☐ None
- ☐ Unknown
- ☐ Other

If other, please specify.

If you have or anticipate to have challenges, please explain them briefly.

Signals and Outbreaks

The following section should be completed by everybody, regardless of whether you have chosen a surveillance system with or without automated outbreak detection tools.

Consider a situation where you have identified an anomalously high number of cases in your surveillance data that commends a signal and triggers public health actions such as verification and outbreak investigation. Please try to answer the following questions irrespective of these signals stemming from an outbreak detection tool or manual inspection.

* Are signals (i.e. from automated outbreak detection tools or manual analysis) followed by or trigger public health actions (i.e. investigation of an outbreak)?

- Yes
- No
- Unknown

If yes, please briefly describe the public health action.

* Is feedback required and given on the public health actions based on the signal?

- Yes
- No
- Unknown

If yes, please describe the form of feedback loop.

* Is the feedback (systematically) collected?

- Yes
- No
- Unknown

* Is the feedback used to evaluate the (automated) outbreak detection signals and the approach (such as tools or manual detection approach) in order to implement changes/improvements?

- Yes
- No
- Unknown

Automated outbreak detection tools

The following section should **only be filled in** in case you have a

- surveillance system with an outbreak detection tool/s

or

- surveillance system with planned implementation of outbreak detection tool/s

Please choose the most advanced and relevant tool used.

What type of tool are you using?

- External software (e.g. SATSCAN)
- Existing open library (e.g. surveillance)
- Self-implemented solution (e.g. R or Stata scripts)
- Unknown
- Other

If other, please specify.

If an external software or an existing library is used, please specify which one.

How complex/ difficult would you rate the use of the tool, i.e. regarding data pre-processing, parameter input, running, output generation?

- Complex/ difficult
- Neither difficult nor simple
- Simple
- Unknown

How demanding is the tool with respect to IT-resources, i.e. servers, memory, computing power, databases?

- Demanding
- Neither demanding nor simple
- Simple
- Unknown

What is the outcome (presentation/communication of the results) of the tool?

- [ ] Dashboard
- [ ] Situation Report
- [ ] List or table
- [ ] Excel or CSV file
- [ ] Unknown
- [ ] Other

If other, please specify.

What statistical method is used?

- Spatial Clustering
- Time Series Outlier
- Fixed Threshold
- Unknown
- Other

If other, please specify.

Is demographic information used for outbreak detection? (i.e. stratifying by age group)

- Yes
- No
- Unknown

How satisfied are you overall with the tool?

Please indicate from very dissatisfied (1 star) to very satisfied (5 stars)	☆☆☆☆☆

Are the generated signals (outbreak warnings) timely enough?

- Agree
- Neither agree nor disagree
- Disagree
- Unknown

Are the generated signals (outbreak warnings) precise (regional, demographic, time) enough?

- Agree
- Neither agree nor disagree
- Disagree
- Unknown

Is the outbreak detection tool/ system too sensitive (i.e. too many generated signals are no true outbreaks)?

- Agree
- Neither agree or disagree
- Disagree
- Unknown

Please share your ideas how your tool can be improved.
