# Supplementary material for: Requirements and Implementation Challenges of Automated Outbreak Detection Systems in the European Union: Mixed Methods Study
Source: JMIR Public Health Surveill. 2026 Sep 15;12:e96576. doi: 10.2196/96576 (PMC13577613; doi:10.2196/96576)
Supplement: Supplementary file 3 [file publichealth-v12-e96576-s002.docx]

Supplementary File 2 – Overview of country-specific source of information and AODS implementation status across 26 countries, 2024

| **Country name** | **Survey** | **Workshop** | **Literature** |  | **No data** | **With systems** | **Pilot** | **Planned implementation** | **No implementation** |
| --- | --- | --- | --- | --- | --- | --- | --- | --- | --- |
| **Austria** | **✓** | **✓** |  |  |  | **✓** |  |  |  |
| **Belgium** | **✓** | **✓** |  |  |  |  |  | **✓** |  |
| **Croatia** | **✓** | **✓** |  |  |  |  |  | **✓** |  |
| **Cyprus** |  |  |  |  | **✓** |  |  |  |  |
| **Czechia** | **✓** |  |  |  |  |  |  |  | **✓** |
| **Denmark** | **✓** | **✓** |  |  |  | **✓** | **✓** |  |  |
| **Estonia** | **✓** | **✓** |  |  |  |  |  |  | **✓** |
| **Finland** | **✓** | **✓** |  |  |  | **✓** | **✓** |  |  |
| **France** |  |  | **✓** |  |  | **✓** |  |  |  |
| **Germany** | **✓** | **✓** |  |  |  | **✓** |  |  |  |
| **Hungary** | **✓** | **✓** |  |  |  |  | **✓** |  |  |
| **Ireland** | **✓** | **✓** |  |  |  |  | **✓** |  |  |
| **Italy** |  |  |  |  | **✓** |  |  |  |  |
| **Latvia** | **✓** | **✓** |  |  |  |  | **✓** |  |  |
| **Lithuania** | **✓** | **✓** |  |  |  |  | **✓** |  |  |
| **Luxembourg** | **✓** |  |  |  |  |  |  |  | **✓** |
| **Malta** | **✓** | **✓** |  |  |  |  | **✓** |  |  |
| **Netherlands** | **✓** | **✓** |  |  |  | **✓** | **✓** |  |  |
| **Norway** |  |  |  |  | **✓** |  |  |  |  |
| **Poland** | **✓** | **✓** |  |  |  |  | **✓** |  |  |
| **Portugal** | **✓** | **✓** |  |  |  |  |  | **✓** |  |
| **Romania** | **✓** |  |  |  |  |  |  | **✓** |  |
| **Slovenia** | **✓** | **✓** |  |  |  |  | **✓** |  |  |
| **Spain** | **✓** |  |  |  |  |  |  |  | **✓** |
| **Sweden** | **✓** |  |  |  |  |  |  | **✓** |  |
| **United Kingdom** |  |  | **✓** |  |  | **✓** |  |  |  |
